# Schizophrenia and Moral Responsibility: A Kantian Essay

**DOI:** 10.1007/s11406-015-9685-4

**Published:** 2016-02-15

**Authors:** Matthé Scholten

**Affiliations:** Department of Philosophy, University of Amsterdam, Oude Turfmarkt 141-147, 1012 CG Amsterdam, The Netherlands

**Keywords:** Schizophrenia, Moral responsibility, Immanuel Kant, Mental disorder, Blame, Psychosis

## Abstract

In this paper, I give a Kantian answer to the question whether and why it would be inappropriate to blame people suffering from mental disorders that fall within the schizophrenia spectrum. I answer this question by reconstructing Kant’s account of mental disorder, in particular his explanation of psychotic symptoms. Kant explains these symptoms in terms of various types of cognitive impairment. I show that this explanation is plausible and discuss Kant’s claim that the unifying feature of the symptoms is the patient’s inability to enter into an exchange of reasons with others. After developing a Kantian Quality of Will Thesis, I analyze some real life cases. Firstly, I argue that delusional patients who are unable to enter into an exchange of epistemic reasons are exempted from doxastic rather than moral responsibility. They are part of the moral community and exonerated from moral blame only if their actions do not express a lack of good will. Secondly, I argue that disorganized patients who are unable to form intentions and to make plans are exempted from moral responsibility because they do not satisfy the conditions for agency.

## Introduction

Since the publication of Peter Strawson’s (1962) seminal article *Freedom and Resentment*, so-called ‘excuses’ and ‘exemptions’ have been at the center of debates about moral responsibility.^1^ Excuses and exemptions are conditions under which it would be inappropriate to blame agents. While inadvertence and physical constraint are paradigm cases of the former, early childhood and mental disorder are paradigm cases of the latter. The topic of this paper is mental disorder. With regard to that type of exempting condition, Strawson left several questions unanswered: Why exactly should we refrain from blaming the mentally disordered? Which mental disorders count as exempting conditions? Do mental disorders exempt in a unified way? Is mental disorder an independent ground of exculpation or merely a sign that one of the standard excusing conditions obtains? These questions have been addressed in the subsequent debate, but the answers given remain controversial.

To many it might come as no surprise that in this debate a Kantian position is still wanting. According to a common picture, the doctrine of transcendental idealism commits Kant to an uncompromising account of freedom that leaves no room for excuses or exemptions.^2^ Some may also be skeptical about whether the philosopher of the a priori provides us with any theoretical instruments for thinking about mental disorder at all. Yet caricatures may be misleading. In fact, Kant wrote extensively and in great detail about mental disorder. But despite its prominence in the *Anthropology* and the *Essay on the Maladies of the Head*, Kant’s account of mental disorder has gone unnoticed even among scholars who devoted their work to Kant’s anthropological works.^3^ This paper is an attempt to fill the gap. My aim is twofold. Firstly, my aim is to show that, contrary to what the common picture suggests, Kant is very attentive to empirical facts about mental disorder and takes such considerations to make a real difference with respect to questions about freedom and responsibility. Secondly, my aim is to make a contribution to the current debate about mental disorder and moral responsibility by showing that Kant provides us with a plausible explanation of psychotic symptoms that is able to explain why mentally disordered patients are exonerated from blame.

Let me introduce a Kantian take on the topic by giving a quote from Kant’s *Lectures on Metaphysics*. Seemingly in accord with the common picture, Kant there asserts that human freedom cannot be curtailed by natural causes. But he immediately adds an important caveat: ‘Only in some cases does he have no power of free choice, e.g., in the most tender childhood, or when he is insane, and in deep sadness, which is however also a kind of insanity’ (V-M_1_ 28: 255).^4^ My concern in this paper is with the claim that people who are ‘insane’ lack the ‘power of free choice.’ Kant is not alone in claiming this. For instance, the fourth edition of the *Diagnostic and Statistical Manual of Mental Disorders* defined mental disorder as a syndrome associated with ‘an important loss of freedom’ (American Psychiatric Organization 1994, xxi).^5^ However right it may intuitively seem to say that those who lack freedom are exempt from moral responsibility, without an answer to the question as to what this lack of freedom precisely consists in, we have no real grasp of why mental disorder would make blame inappropriate.

Philosophers have sometimes given rather general answers to questions about the accountability of the mentally disordered, but closer attention reveals that the answer to the question whether and why mental disorder makes blame inappropriate depends on the nature of the specific mental disorder under consideration.^6^ For that reason, I focus exclusively on mental disorders that fall within the schizophrenia spectrum. The symptoms that define this spectrum are commonly grouped into ‘positive’ and ‘negative’ symptoms. While symptoms such as delusions, hallucinations, disorganized thought and disorganized behavior are counted among the former, symptoms such as flattening of affect, poverty of action and poverty of speech are counted among the latter (American Psychiatric Organization 2013, 87). All the claims I make in this paper are restricted to patients in the acute phase of their illness showing at least one of the positive symptoms.

The paper is structured as follows. In the following section, I draw a distinction between excuses and exemptions and show that in Kant’s view disorders involving psychotic symptoms qualify as exempting conditions. I then reconstruct Kant’s explanation of psychotic symptoms. Subsequently, I analyze Kant’s claim that the general characteristic of psychotic symptoms is the impairment of the patient’s ability to make public use of reason. To prepare the ground for the analysis of some real life cases in the final sections of this paper, I develop a Kantian Quality of Will Thesis. In the section ‘Epistemic Incapacity,’ I discuss three cases of delusional patients who act from ignorance about the nature and circumstances of their actions and explain why they should be exonerated from blame. In the section ‘Practical Incapacity,’ I discuss a case of a disorganized patient who fails to satisfy the requirements of agency and explain why this patient should be exempted from moral responsibility.

## Excuses and Exemptions

Suppose an agent is accused of performing some morally objectionable action. In that situation, the agent can object to being blamed in roughly two ways. Firstly, she can provide a justification for the action. In this case, she accepts responsibility for the action yet pleads that, all things considered, she did what was morally right. Alternatively, she can provide an excuse for the action. In that case, she acknowledges that the action is morally wrong yet pleads that she is not morally responsible for it.^7^ This broad category of excuses falls into two sub-classes: excuses in the narrow sense and exemptions. Excuses (in the narrow sense) and exemptions both defeat the claim that an agent is morally responsible for a specific action, but they do so in different ways. Excuses do so by showing that certain features pertaining to the *action* and its etiology block the agent’s responsibility for the action. Since excuses depend only on features of the specific action under consideration, they do not give us reason to stop treating the agent as a potential candidate for moral blame. Exemptions, on the other hand, defeat the claim that an agent is morally responsible for a specific action by showing that the *agent* has certain features that give us reason to refrain from treating her as a potential candidate for moral blame. According to an influential view, agents are properly exempted from moral responsibility insofar as they are unable to understand and to respond to moral demands.^8^ Although exemptions typically function more globally than excuses, it is important to note that exemptions need not be permanent or even long-lasting. For example, it might very well be that a person lacks the relevant abilities during a relatively short psychotic episode, but regains those abilities upon recovery. Such a person may thus be exempted from moral responsibility with regard to a specific action she performed during the psychotic episode, but not with regard to later actions.

Excuses and exemptions are often supposed to be radically different pleas.^9^ However, as I will show in the section ‘Epistemic Incapacity,’ only a combination of excusing and exempting conditions can explain why blame seems inappropriate in some complex cases. To anticipate, in these complex cases, agents are primarily exempted from doxastic responsibility, and they are morally excused only given that exemption. In such cases, the agent is thus neither simply exempted nor simply excused. To make this explicit, I will say that such agents are ‘exonerated from blame.’ Since my discussion of these complex cases relies on an account of excuses, I will give a brief typology of excuses here.^10^ Suppose agent *S* is called to account for some act or omission *A* under a description *x*. She can excuse herself in the following ways:

1. *Inadvertence*: even if *A* was intentional under some description, it was not intentional under description *x*. (For example, *S* accidently slapped someone in the face when putting on her jacket.)
2. *Unintentional bodily movement*: *A* was not intentional under any description. (*S* sneezed or *S* was pushed.)
3. *Physical constraint*: *S* omitted *A*, but the omission is solely due to the fact that the necessary means to *A* were not available to *S*. (*S* did not show up at an appointment because her car broke down unexpectedly, or because her legs were suddenly paralyzed.)
4. *Coercion and necessity*: *A* was intentional under description *x*, but *S* performed *A* only in order to avoid some great harm. (*S* handed over the money from the cash register in order to avoid being shot; *S* shoved someone off a floating plank in order to save herself from drowning.)

Later, I will develop a Kantian Quality of Will Thesis that supports these excuses, but for now, I simply assume this typology to be unproblematic. What interests me here is that in Kant’s view there is another condition that can make blame inappropriate: ‘If someone has intentionally caused an accident, the question arises whether he is liable and to what extent; consequently, the first thing that must be determined is whether or not he was mad at the time’ (Anth 7: 213). Although Kant is primarily concerned with legal liability here, the passage gives us an important clue about the conditions for moral responsibility. The passage suggests that if it would turn out that the agent was ‘mad’ at the time, the agent would not be liable for the harm she caused. From the context, it is clear that Kant refers to patients suffering from the type of mental disorder that he labels ‘mental derangement.’ As a first step to understanding why the mentally deranged are exonerated from blame, I will therefore reconstruct Kant’s explanation of the symptoms of mental derangement.

## Kant’s Explanation of Psychotic Symptoms

Given that the term ‘schizophrenia’ was coined by Bleuler in the beginning of the 20th century, it would be anachronistic to say that Kant provides us with an account of schizophrenia. In this section, however, I will show that the symptoms that Kant discusses under the header ‘mental derangement’ correspond to what we nowadays regard as the positive symptoms of schizophrenia.^11^ Kant explains mental derangement primarily in terms of the impairment of cognitive faculties, but this is not to deny that it is caused by physical processes in the brain. In fact, Kant thinks that the ‘roots’ of mental derangement ‘lie in the body’ (VKK 2: 270).^12^ According to Kant’s account of the causal genesis of the disorder, mental derangement occurs when hereditary biological factors are triggered by social circumstances (Anth 7: 217; VKK 2: 269).

One of Kant’s reasons for focusing on the ‘appearances in the mind’ (VKK 2: 270) rather than on the physical causes of mental disorder is his awareness of the limits of his scientific competence. But more importantly for our aims, Kant holds that whether a mentally disordered agent can be held responsible for an action is ‘a wholly psychological question’; to be precise, it is a ‘question of whether the accused at the time of his act was in possession of his natural faculties of understanding and judgment’ (Anth 7: 213f.). Since facts about psychological states and capacities of agents bear on questions of responsibility, close observation of the ‘appearances in the mind’ of the disorder is a first step to answering the question why mental disorder can make blame inappropriate.^13^

The ingenious aspect of Kant’s discussion of mental derangement is his explanation of its symptoms in terms of the impairment of the cognitive faculties necessary for experiential cognition that he laid out in the *Critique of Pure Reason*. Kant distinguishes four different types of mental derangement. They are related to the impairment of respectively the faculty of the understanding, the power of imagination, the power of judgment and the faculty of reason. I will discuss them in a somewhat different order. I will first discuss two symptoms that are associated with the so-called paranoid type of schizophrenia and then proceed to two symptoms associated with the so-called disorganized type.

Let us start with persecutory and referential delusions, both of which are well-known symptoms of the schizophrenia spectrum. Kant is familiar with these symptoms. He observes that some mentally deranged patients ‘believe they are surrounded by enemies everywhere, [and] consider all glances, words, and otherwise indifferent actions of others as aimed against them personally and as traps set for them’ (Anth 7: 215). He assigns the symptoms to a type of mental derangement that he labels ‘*dementia*’ and points out that they are best explained by an impairment of the power of imagination (Anth 7: 215).^14^

In the *Critique of Pure Reason*, Kant defines the power of imagination as ‘the faculty for representing an object even without its presence in intuition’ (B151). In its reproductive function, it enables us not only to bring to mind past experiences but also to make up representations of non-existent objects. The distinguishing feature of the reproductive imagination is the fact that its activity is subject only to psychological laws of association (B152). This characteristic enables us to distinguish representations that correspond to objects given in intuition from representations that lack such corresponding objects. These differ not so much in their content or intensity as in the way they are connected: whereas the former are connected primarily through their content under subjective laws of association, the latter are synthesized into a unified experience by the formal and objective laws of the understanding.^15^ In Kant’s view, persecutory and referential delusions are explained by the patient’s inability to distinguish between these two types of representation. Due to this cognitive impairment, the delusional patient experiences the objects of her self-made representations as if they were really given: ‘owing to the falsely inventive power of imagination, self-made representations are regarded as perceptions’ (Anth 7: 215).

A further class of symptoms that psychiatrists count among the key symptoms of the schizophrenia spectrum is the class of so-called ‘bizarre delusions.’ Familiar kinds of bizarre delusions recounted in contemporary psychiatric literature are thought insertion and delusions of control. Patients experiencing thought insertion may report things like, ‘The thoughts of Aemonn Andrews come into my mind. He treats my mind like a screen and flashes his thoughts on it like you flash a picture’ (Frith and Johnstone 2003, 36).^16^ Patients experiencing delusions of control may say things like, ‘It is my hand and arm that move, and my fingers pick up the pen, but I don’t control them’ (Frith and Johnstone 2003, 37). In this context, Kant refers to patients who claim to have comprehended ‘the mystery of the Trinity’ or to have invented things like ‘the squaring of the circle’ or ‘perpetual movement’ (Anth 7: 215f.). However different from thought insertion and delusions of control these symptoms may seem at first sight, some important similarities will become apparent if we assess Kant’s explanation of the symptoms.

Kant assigns the symptoms just named to a form of mental derangement that he refers to as ‘*vesania*.’ *Vesania* consists in ‘the sickness of a deranged *reason*’ (Anth 7: 215). In the *Critique of Pure Reason*, Kant defines reason as ‘[the faculty] of drawing inferences mediately,’ that is, as the ability to draw conclusions from premises by using syllogisms (A299/B355). In this function, reason is guided by the principle of non-contradiction (A150/B189f.). But Kant uses the word ‘reason’ in a second way. Regularly, he uses ‘rational cognition’ [*Vernunfterkenntnis*] to refer to knowledge attainable a priori, that is, to knowledge of the conditions for the possibility of experience. In keeping with this twofold use, reason demarcates the realm of meaningful propositions in two ways. Firstly, reason restricts the realm of meaningful propositions to logically possible propositions, that is, to propositions that conform to the principle of non-contradiction. This restriction rules out from the realm of meaningful propositions the proposition that God is both one and three at the same time and in the same respect. Secondly, reason restricts the realm of meaningful propositions to empirically possible propositions, that is, to propositions that conform to the forms of intuition (i.e., linear time and Euclidean space) as well as to the rules of the understanding (roughly the general laws of pure Newtonian natural science). Whereas the former aspect of this restriction rules out the possibility of squaring the circle, the latter aspect, in conjunction with some basic facts about the world, precludes the possibility of perpetual movement.

Kant’s explanation of the symptoms of *vesania* in terms of an impairment of reason adequately captures the distinguishing feature of bizarre delusions. Persecutory and referential delusions are generally logically and empirically possible – though of course not therefore true. Bizarre delusions, on the other hand, are defined as beliefs that are ‘clearly implausible and not understandable to same-culture peers’ or as beliefs the content of which ‘the person’s culture would regard as physically impossible’ (American Psychiatric Organization 2013, 87, 819). True, the writers of the *DSM* neither purport nor want to be transcendental philosophers, but the similarity between the beliefs referred to by Kant and the beliefs that today’s psychiatrists call ‘bizarre delusions’ is clear: both express ideas that are (or that same-culture peers take to be) logically or empirically impossible.

I now turn to the symptoms associated with the disorganized type of schizophrenia. A first key symptom is a form of disorganized speech commonly referred to as ‘derailment’ or ‘loose association.’ In a conversation, individuals exhibiting this form of disorganized speech may suddenly switch from one topic to another (American Psychiatric Organization 2013, 88). Being acquainted with this symptom, Kant assigns it to a class of mental derangement that he labels ‘*insania*.’ *Insania* is due to a ‘deranged *power of judgment*’ (Anth 7: 215). In the *Critique of the Power of Judgment*, Kant defines the power of judgment as ‘the faculty for thinking of the particular as contained under the universal’ (KU 5: 179). To take a concrete example, the power of judgment enables us to subsume particular dogs under the general concept ‘dog’ and to subsume the concept ‘dog,’ together with other concepts like ‘cat,’ ‘cow,’ and ‘fox,’ under the more general concept ‘animal.’ Kant points out that due to an impairment of this cognitive capacity ‘the mind is seized by analogies that are confused with concepts of similar things, and thus the power of imagination, in a play resembling understanding, conjures up the connection of disparate things as universal’ (Anth 7: 215).

As an illustration of this form of disorganized speech, consider the following example. In a study of nearly 300 schizophrenic patients conducted by Christopher Frith and his colleagues, different groups were asked to perform a verbal fluency task. The task was to name animals. Whereas the group of patients with poverty of speech tended to commit errors of omission, the group of patients with the disorganization syndrome tended to make errors of commission. As an example of the latter type of error, Frith and Johnstone quote a patient who produced the sequence, ‘emu, duck, swan, lake, Loch Ness monster, bacon …’ (2003, 63). This strongly suggests that Kant’s explanation of derailment and loose association in terms of an impairment of the power of judgment is correct.

A last key symptom of disorganized schizophrenia is commonly referred to as ‘word salad.’ In contrast to derailment, the incoherence in word salad occurs within rather than between clauses, which results in speech that is ‘so severely disorganized that it is nearly incomprehensible’ (American Psychiatric Organization 2013, 88, 823). Kant is familiar with the symptom and observes that some patients speak in such a way that ‘no one grasps what they actually wanted to say’ (Anth 7: 215). Assigning the symptom to a class of mental derangement labeled ‘*amentia*,’ he points out that the symptom is explained by ‘the inability to bring one’s representation into even the coherence necessary for experience’ (Anth 7: 214). Since the understanding is constitutive of experience by synthesizing representations, this suggests that Kant takes word salad to be explained by an impairment of the understanding.

In the first *Critique*, Kant defines the understanding as ‘a faculty for judging’ (A69/B94). For Kant, to judge is to join two concepts together in such a way that one forms a proposition that is either true or false. ‘Categorical’ judgments provide the typical example. In a categorical judgment, a predicate is ascribed to a subject by means of the copula ‘is’ (e.g., ‘This table is brown’). ‘Hypothetical’ judgments of the form ‘if *x*, then *y*’ provide another example. To see how the impairment of the patient’s ability to judge explains the word salad, consider the following case report. In a study in which patients exhibiting the symptom were asked to explain the difference between courage and recklessness, a patient answered,

Courage can only arise from that which he is himself, therefore I can [pause] on the contrary is recklessness, he wants to establish something, launch something, but he doesn’t really have it inside, consequently the truth doesn’t fully stand behind it, recklessness, that is risky things, yes, as opposed to courage.^17^

When trying to explain the difference between courage and recklessness, most of us would start either by giving definitions (‘Courage is…, whereas recklessness is…’) or by giving conditional statements (‘If someone is courageous, she…; if someone is reckless, she…’). The patient’s inability to distinguish between the two concepts and the resulting word salad can thus plausibly be explained by an impaired ability to employ categorical and hypothetical judgments.

## Mental Derangement and the Public Use of Reason

To wrap up the results of the previous section, on Kant’s view mental derangement can involve four types of cognitive impairment. It can involve an impairment (i) of the power of imagination, in particular of the capacity to distinguish ‘self-made’ representations from representations that correspond to objects in intuition, (ii) of the faculty of reason, as the capacity to distinguish meaningful from nonsensical propositions, (iii) of the power of judgment, as the capacity to subsume particulars under general concepts or (iv) of the faculty of the understanding, as the capacity to judge.

Interestingly, Kant claims that these four types of mental derangement have something in common: ‘The only universal characteristic of madness is the loss of common sense (*sensus communis*) and its replacement with logical private sense (*sensus privatus*)’ (Anth 7: 219). Kant’s claim here is not that the mentally disordered lack common sense. As noted by O’Neill (1989, 25, 45), ‘common sense’ is misleading as a translation of the German ‘*Gemeinsinn*’ or the Latin ‘*sensus communis*.’ The fact that Kant contrasts *sensus communis* with *sensus privatus* suggests that ‘*sensus communis*’ refers to a sense that is public. This is confirmed by the following famous passage:

By ‘*sensus communis*’ […] should be understood the idea of a communal sense, i.e., a faculty for judging that in its reflection takes account (*a priori*) of everyone else’s way of representing in thought in order as it were to hold its judgment up to human reason as a whole and thereby avoid illusion (KU 5: 293).

In other words, the loss of *sensus communis* on the part of the mentally deranged refers to an impairment of their ability to critically reflect on their beliefs by asking whether their beliefs could be endorsed by all others: it is an impairment of the ability to ‘reflect on one’s own judgment from *a universal standpoint*,’ a standpoint that can only be acquired by ‘putting oneself into the standpoint of others’ (KU 5: 295). In his essay *What is Enlightenment?*, Kant refers to this ability to address oneself to ‘the world at large’ as the ability to make public use of reason (WA 8: 38, 57). So in a word, the unifying feature of psychotic symptoms is the patient’s impaired ability to make public use of reason.

This impairment is no minor thing. According to Kant, it is ‘a subjectively necessary touchstone of the correctness of our judgments’ that we ‘restrain our understanding by the *understanding of others*, […] judging *publicly* with our private representations’ (Anth 7: 219). If our ability to enter into an exchange of reasons with others enables us to subject our beliefs to rational critique, then the patient’s inability to make public use of reason entails an impairment of her ability to reflect on her beliefs in a critical manner. Beliefs can be ‘fixed’ either in the sense that they are not susceptible to rational argumentation or in the sense that they cannot be revised in the light of empirical evidence. The immunity of beliefs to critical revision plays an important role in the diagnosis of schizophrenia. For example, it is considered a defining feature of delusions that they are held despite ‘incontrovertible and obvious proof or evidence to the contrary’ (American Psychiatric Organization 2013, 819).

At the extreme, the loss of public sense could give way to illusion in such a way that the mentally deranged person experiences things that others do not experience. Kant gives the example of a person who ‘in broad daylight sees a light burning on his table which, however, another person standing nearby does not see, or hears a voice that no one else hears’ (Anth 7: 219). In passing, Kant here touches upon another key symptom of schizophrenia, namely hallucination.^18^ While hallucination is the clearest case in which a person loses a sense of a shared world, Kant claims that the loss of a shared world is a general aspect of mental derangement. The mentally deranged person, he notes, ‘is abandoned to a play of thoughts in which he sees, acts, and judges, not in a common world, but rather in his own world’ (Anth 7: 219).

The impairment of the ability to make public use of reason and the associated loss of a shared world are factors that have implications for the attitude we take toward the mentally deranged. This is most obvious in the epistemic domain. Even though it may sometimes be extremely hard to convince sane people of the truth, their beliefs are, at least in principle, susceptible to rational persuasion. For that reason, the fact that it is extremely hard to convince a sane person of the truth gives us no reason to stop trying (though perhaps the fact that we get tired of it does). On the other hand, Kant insists that we should not try to convince a deluded person of the falsity of her beliefs; it may be better simply to go along with the patient (VKK 2: 270). This is because trying to convince the patient of the incorrectness of her representation would be much like trying to assure yourself that you are not in pain when in fact you are crying out with it: ‘It would be in vain to set rational arguments against a sensation or that representation which resembles the latter in strength, since the senses provide a far greater conviction regarding actual things than an inference of reason’ (VKK 2: 264f.).

Similar considerations hold for the moral domain. When in the *Essay on the Maladies of the Head* Kant winds up a discussion of some ‘frailties of the head’ that are best described as character flaws and turns to the discussion of mental derangement (here labeled ‘the disturbed mind’), he remarks,

I come now from the frailties of the head which are despised and scoffed at to those which one generally looks upon with pity, or from those which do not suspend civil community to those in which official care provision takes an interest and for whom it makes arrangements (VKK 2: 263).

When it comes to complying with moral principles, everyone has their shortcomings. But we see a sane person’s shortcomings as something to be ‘scoffed at,’ as something for which she deserves blame. Adopting this attitude, we see her as a potential candidate for ‘civil community,’ that is, as someone with whom we can enter into an exchange of publicly accessible reasons. By contrast, since the mentally deranged are unable to enter into an exchange of publicly accessible reasons, we look upon their shortcomings with pity; their misfortune evokes sorrow rather than contempt and calls for care rather than blame.^19^

## Quality of Will

In the final sections of this paper, I will provide an answer to the question whether and why it would be inappropriate to blame people suffering from schizophrenia. To prepare the ground for this analysis, I develop a Kantian Quality of Will Thesis in this section. I do so by focusing on the excuse that is most relevant to the explanation of why it would be inappropriate to blame schizophrenic patients, namely the excuse of inadvertence.

On a Kantian view, maxims are the primary objects of moral assessment. Kant defines a maxim as ‘the subjective principle of acting’ (GMS 4: 420n.). Maxims have the following form: ‘If I am in circumstances of the type *C*, then I perform an action of the type *A*’.^20^ There is scholarly disagreement about the level of generality of maxims, in particular about whether they should be construed as specific intentions or as general policies.^21^ Fortunately, this question need not bother us here, since for the purpose of moral assessment both levels matter. Usually maxims form a hierarchical structure in which more specific intentions are connected to more general policies by principles of consistency. If maxims are ordered in that way, particular actions express both the agent’s specific intentions and her general policies. But intentions and policies may also come apart. Typically, negligent actions express only general policies and actions that appear to be ‘out of character’ express only specific intentions. To reflect both levels of generality, I shall say that an action manifests good will if it expresses morally agreeable intentions *and* attitudes and that it manifests a lack of good will if it expresses morally objectionable intentions *or* attitudes. By ‘attitude,’ I mean a disposition of an agent that is under her voluntary control in the sense she can alter it by adopting a different policy.^22^

Kant introduces his Quality of Will Thesis in his discussion of moral worth in *Groundwork I*. There he makes the important observation that the concept of the will ‘always takes first place in estimating the total worth of our actions’ (GMS 4: 397). He elaborates on this idea in a famous passage, which I will quote at length:

A good will is not good because of what it effects or accomplishes […], but only because of its volition, that is, it is good in itself […]. Even if, by a special disfavor of fortune or by the niggardly provision of a stepmotherly nature, this will should wholly lack the capacity to carry out its purpose – if with its greatest efforts it should yet achieve nothing and only the good will were left (not, of course, as a mere wish but as the summoning of all means insofar as they are in our control) – then, like a jewel, it would still shine by itself, as something that has its full worth in itself. Usefulness or fruitlessness can neither add anything to this worth nor take anything away from it (GMS 4: 394).

I take it that an action’s moral worth determines whether a person is praise- or blameworthy for performing the action and, furthermore, that to say that a person’s will has ‘full worth in itself’ is to say that she is not blameworthy. Read in this light, Kant’s claim is that if an agent adopted a maxim that she could at the same time will as a universal law and truly did all she could to translate that maxim into action, she could reasonably object if others blame her for her omission. She can reasonably object because her omission does not manifest a lack of good will.

Without adducing further arguments, it is clear that this Kantian Quality of Will Thesis supports the excuse of physical constraint. Since unintentional bodily movements obviously do not express the agent’s will, it evidently supports the excuse of unintentional bodily movement as well.^23^ While assuming that it supports the excuse of coercion and necessity, I will argue that the Kantian Quality of Will Thesis supports the excuse of inadvertence.^24^

I will do so by discussing three examples. Before I turn to these examples, a brief comment is in place. It is well known that, other than many contemporary moral theories, Kant’s moral theory contains no direct criterion for the rightness of actions: Kant’s universalization test applies to maxims rather than actions. Nevertheless, Kant not only explicitly claims that the Categorical Imperative renders actions morally permissible, morally impermissible or morally obligatory (MS 6: 222; GMS 4: 439), but he also holds that an action can be ‘in accordance with duty’ even if it is not based on the right maxim. This is not inconsistent. Korsgaard (1996b, 47, 47n.) shows that a Kantian criterion of rightness of actions can be defined in a derivative way. Building on her account, I will assume the following criterion of moral permissibility for actions in my discussion of the examples: action *A* is morally permissible in circumstances *C* just when an agent who acts on the right maxim would, under favorable circumstances, perform *A* in *C*. The clause ‘under favorable circumstances’ serves to rule out conditions such as ignorance, physical constraint or weakness of will. This clause is necessary because it is intuitively clear that, under these conditions, agents may not do the right thing even if they adopted the right maxim.

We can now proceed to the examples. Suppose that Jane and Joe are doctors who can save your life by giving you some treatment.^25^ Let us stipulate that the doctors ought to act on a life-saving maxim. Consider the first example:

*Jane* wants to save your life and therefore gives you treatment *x*, which she believes and on sufficient evidence will most likely save your life. It turns out that the treatment saves your life.

I assume no one will think that Jane deserves blame for giving you the treatment. Now suppose there is an alternative situation in which you have a slightly different bodily constitution. However, there is no evidence for this. This time you receive treatment from Joe:

*Joe* wants to save your life and therefore gives you treatment *x*, which he believes and on sufficient evidence will most likely save your life. It turns out that the treatment kills you.

Joe killed you. Since this is clearly not the sort of action that an agent who acts on the right maxim would, under favorable conditions, perform, Joe’s action is morally impermissible.^26^ Even so, it seems unfair to blame Joe for doing what he did. After all, there seems to be no morally significant difference between Jane and Joe. The only difference is that Jane’s belief was true while Joe’s was not, but Joe was justified in holding the belief and would have acted in a morally permitted way if his belief were true. So why blame Joe given that we do not blame Jane? Our intuition that Joe should not be blamed can be explained by Kant’s observation that the will ‘always takes first place in estimating the total worth of our actions’: even if Joe killed you, his action did not manifest a lack of good will. For that reason, Joe has a valid excuse and hence we should refrain from blaming him.

Now imagine a situation in which you have the same bodily constitution as in the previous one, but now there is clear evidence for that fact. This time you receive treatment from Jack:

*Jack* wants to save your life and therefore he gives you treatment *x*, which he believes will save your life but on sufficient evidence will most likely kill you. It turns out that the treatment kills you.

Jack killed you. Here, too, the action is morally impermissible according to the assumed criterion of rightness. However, Jack would have acted in a morally permitted way if his beliefs were true. So given what we know about Jack, his action does not manifest morally objectionable intentions. That is a good reason for not accusing him of murder. But now return to Joe. Part of the reason why we think that he should not be blamed is that we assume that if there were sufficient evidence about your bodily constitution available to him, he would not have given you treatment *x*. Jack on the other hand gave you treatment *x* despite the availability of such evidence. Suppose that his failure to respond to the evidence was due to his carelessness. In that case, Jack would have showed a lack of good will by culpably failing to adopt a policy that would enable him to respond to morally significant evidence. So even if his action does not directly manifest morally objectionable intentions, Jack is not excused for killing you. His ignorance alone is not sufficient to get him off the hook.

From these examples, we can infer the following Quality of Will Test for actions done from ignorance:

*Quality of Will Test*: Agent *S* is excused for performing a morally wrong action *A* if,

(i) *S* had false beliefs about the nature or circumstances of *A;*
(ii) these false beliefs feature in a rationalizing explanation of *A;*
(iii) *A* would be morally permitted or excused if *S*’s beliefs about the nature and circumstances of *A* were true; and
(iv) *S* is not culpable for having these false beliefs

The second condition ensures that the agent acted on the false beliefs. The third condition constitutes the kernel of this Quality of Will Test. In conjunction with the other conditions, it captures the Kantian intuition that we should judge people by the quality of will that their actions manifest rather than directly by their actions.

## Epistemic Incapacity

By discussing three real life cases, I will now attempt to show that this test can explain why schizophrenic patients of the paranoid type are exonerated from blame. Before I turn to the case descriptions, I have to add three caveats. Firstly, apart from having impaired epistemic capacities, arguably some persons suffering from paranoid schizophrenia fall short of the standards of practical rationality. For the sake of clarity, I will focus exclusively on epistemic capacities here, deferring a discussion of practical rational capacities to the following section. In light of this, I will assume that the patients in the case descriptions are sufficiently practically rational both in the sense that they have the ability to assess the moral permissibility of what they believe to be doing and in the sense that they have the ability to select the appropriate means for the ends they set.^27^ Secondly, since the focus of this paper lies on the conditions for blame, the cases I describe in the current and the following section involve immoral actions, some of which are violent. However, I do not assume that schizophrenic patients are dangerous.^28^ Studies show that even though there is an association between schizophrenia and violence, this association is small (Walsh et al. 2002). Lastly, my focus will be on relatively clear cases. By doing so, I do not mean to deny that there are complex cases. In fact, I think that most real life cases will be much more complicated than the cases I discuss in this paper. But my hope is that our ability to pass judgment on hard cases will be improved by focusing first on the principles that enable us to decide on relatively clear cases.

Let us turn to the first case. It is a case of a patient suffering from persecutory delusions:

*Case A* is a 40-year-old man who is married and who has three children. He believed that he was overheard making disparaging remarks about drug dealers one day and that this conversation was reported back to the drug ‘mafia,’ who concluded that he must be a police informer. He began to notice that he was being followed by groups of young men who operated from a fleet of cars and believed that these men wanted to catch and kill him. The idea created such panic that Case A ran away from home.^29^

The second case is a case of the delusional syndrome Capgras, a relatively rare syndrome associated with paranoid schizophrenia. The delusions are bizarre:

*Case B*, a 48 year-old male university undergraduate, was convinced that his mother had been replaced by an exact replica, ‘Beelzebub,’ the devil. ‘Beelzebub’ was trying to kill him and destroy the world. Case B wanted to prevent ‘Beelzebub’ from doing that and carried out a prolonged and violent attack on his mother.^30^

The third case involves command hallucinations:

*Kevin* reported that voices commanded him to harm himself and others. He believed that the voices were much stronger and much more able to harm him than he was able to harm them. Kevin reported that he had hit people in response to the voices on three or four occasions.^31^

To answer the question of whether these agents are blameworthy, we must determine whether their actions express a lack of good will. This can be done by subjecting the cases to the Quality of Will Test for actions done from ignorance developed in the previous section. To see that condition (iii), the kernel of the test, is in accord with the way Kant deals with delusional symptoms, consider his remark that delusional patients are usually so ‘astute’ in interpreting their delusions, ‘that, if only the data were true, we would have to pay due honor to their understanding’ (Anth 7: 215).

Since Case A, Case B and Kevin all have false beliefs about the nature or circumstances of their actions, condition (i) is satisfied in each case. Furthermore, it seems safe to say that in each case the agent’s action is explained by the fact that the agent had these false beliefs. That means that condition (ii) is satisfied as well. I will now investigate whether condition (iii) is satisfied. I start with Case B, because even if it is an extreme case, it is also the most straightforward one. Assuming that it is not morally wrong to kill the devil, Case B would have acted in a morally permitted way if his beliefs were true. Condition (iii) is thus satisfied.

Now turn to Case A. I start from the assumption that it is morally wrong to abandon those to whom one has special obligations of care and assistance. Unlike the moral status of Case B’s action, the nature of Case A’s action does not change if we imagine the counterfactual situation in which his beliefs are true: in that situation, Case A would still neglect his special obligations by running away from home. On the other hand, Case A would run away from home only to avoid some great harm, namely the harm of being killed. Accordingly, if Case A’s beliefs were true, his action would fall under the excuse of necessity. With respect to his action, then, condition (iii) is satisfied.

Unlike Case A and Case B, Kevin has false experiences rather than just false beliefs. But like in the previous case, the nature of Kevin’s actions does not change when we imagine the counterfactual situation in which his beliefs are true. In that situation, Kevin would still be hitting innocent people. Yet much like the cashier who hands over the money from the cash register when being held at gunpoint, Kevin complied with the commanding voices only because these voices threatened to harm him in the case of non-compliance. So if Kevin’s beliefs were true, his actions would fall under the excuse of coercion. That means that condition (iii) is satisfied.

Up to now, it may seem as if we should refrain from blaming Case A, Case B and Kevin simply because they have a valid moral excuse. Maybe it is for this reason that Joel Feinberg claims that ‘mental disorder should not itself be an independent ground of exculpation, but only a sign that one of the traditional standard grounds – compulsion, ignorance of fact, or excusable ignorance of law – may apply’ (1970, 272). That is not quite right. Remember that the mere fact that Jack wanted to save your life and believed that giving you treatment *x* was the best available means to attain that end did not get him off the hook. In Jack’s case, condition (iv) is not satisfied. Likewise, in the absence of any standard epistemic excuse, non-psychiatric agents who would have the same desires and beliefs as Case A, Case B or Kevin (even if it may be hard to imagine such cases) would not be morally excused.

Case A, Case B and Kevin do not have any standard epistemic excuse; the available evidence was not contradictory and neither did pressure of time prevent them from assessing the evidence. However, on Kant’s analysis, the symptoms these persons exhibit is due to an impairment of their cognitive faculties, to wit the faculties of imagination and reason. Due to this cognitive impairment, they lack the ability to enter into a mutual exchange of epistemic reasons and to revise their beliefs about the nature and circumstances of their actions in the light of proof and evidence. If, at the time of their action, Case A, Case B and Kevin lacked the ability to respond to epistemic demands, they were exempt from doxastic responsibility. For that reason, they cannot be held responsible for their ignorance. Condition (iv) is thus satisfied. Accordingly, inasmuch as its exculpating force does not reduce to any of the standard excuses, mental disorder is an independent ground of exculpation.

Although an independent ground, mental disorder is not always a sufficient ground of exculpation. On the assumption that patients suffering from mental disorders that involve hallucinations or delusional beliefs about the world are in possession of the necessary practical and normative capacities, they are not exempted from moral responsibility and hence subject to a demand of good will. They are exonerated from blame only on the condition that their actions would be either morally permitted or excused if their beliefs were true. A person who has the encapsulated delusion that his colleagues tap his phone calls can thus still be held responsible for insulting his mother in law on her birthday party. After all, even if his beliefs were true, his action would not be justified or excused.

## Practical Incapacity

In this section, I argue that on a Kantian view people who suffer from mental disorders in the schizophrenia spectrum that involve grossly disorganized thought and behavior are directly exempted from moral responsibility on the grounds that they do not satisfy the requirements for moral agency. Consider Robert’s case:

*Robert Bradstone* was a 32-year-old maintenance worker with a history of schizophrenia. On one evening, he destroyed a display of television sets in a local electronics shop. The police officers who arrested him reported that he made no effort to steal any of the sets and did not try to escape when they arrived. He just stood there mumbling incoherently to himself and gave unintelligible answers to their questions. When Robert was brought to a psychiatric hospital and some residents asked him what brought him there, he answered: ‘Four o’clock is too early. Birds are no goddamn help. Just ask Paul, if you don’t believe me.’^32^

Robert exhibits two symptoms of the schizophrenia spectrum, namely grossly disorganized speech that amounts to word salad and grossly disorganized behavior. Disorganized behavior manifests itself in problems with any form of goal directed behavior, leading to difficulties in performing activities of daily living (American Psychiatric Organization 2013, 88). To refresh our memory, on Kant’s analysis, word salad is due to an impairment of the understanding (i.e., of the ability to make judgments). In what follows, I show that this impairment may also account for disorganized behavior.

For Kant, there are constitutive constraints on agency. A very important constraint is expressed by the Principle of Hypothetical Imperatives, which runs as follows: ‘Whoever wills the end also wills (insofar as reason has decisive influence on his actions) the indispensably necessary means to it that are within his power’ (GMS 4: 417). Suppose you want to have a nice dinner but that the very thought of having to mess around with pots and pans makes you decide to take an unappetizing ready-made meal from the fridge and put it in the microwave. Kant’s point is that in such a situation you did not really want to have a nice dinner. Rather, you wanted to laze around on the couch. In short, his point is that agents must comply with the Principle of Hypothetical Imperatives in order to will at all. Notwithstanding Kant’s assertion that the principle is ‘analytic,’ the clause in parenthesis leaves open the possibility of weakness of the will: it is possible for agents to will some end yet (irrational as it is) not will the necessary means to that end.

Of course, Kant’s claim is not that we explicitly formulate a hypothetical judgment when acting, filling in the blanks with our end and the available means. Usually the principle is a background condition that is implicitly understood. Nevertheless, it goes without saying that the Principle of Hypothetical Imperatives can provide guidance only to agents who have the ability to make hypothetical judgments. This is important with regard to Robert’s case. In Kant’s view, the word salad that Robert exhibits is explained by an impaired ability to make judgments. If Robert’s ability to make hypothetical judgments is impaired, he is unable to guide his behavior in the light of the Principle of Hypothetical Imperatives and, by implication, it must be hard for us to conceive of his actions as expressing any specific intentions at all. That seems an adequate account of the case: the fact that Robert smashed the shop window can hardly be conceived of as a means to steal the television sets, but neither can it be conceived of as an act of vandalism or even as a case of weak will.

Having determined that Robert’s actions fail to express specific intentions, let us assess whether they express any attitudes on his part. According to Kant, actions bring with them another form of commitment. In Kant’s view, agents necessarily act on maxims, and that means that choosing to perform an action of the type *A* in circumstances of the type *C* rationally commits one to adopting a policy of the form, ‘If I am in circumstances of the type *C*, then I perform an action of the type *A*.’ The reason is that if in *C* one judges reason *R* to be sufficient for *A*-ing, then on pain of inconsistency one must judge *R* to be sufficient for *A*-ing in all circumstances relevantly similar to *C*. It requires two cognitive capacities to adopt policies of this kind, namely the capacity to make hypothetical judgments (which is a function of understanding) and the capacity to subsume particular actions and situations under general types (which is a function of the power of judgment). Kant leaves open the possibility that agents accept a general policy yet make an exception for themselves by saying to themselves, ‘all right then, but only for this once’ (cf. GMS 4: 424). If they do so, they are inconsistent. Although we need not always explicitly commit ourselves to a policy by filling in situation-types and action-types in a maxim of a hypothetical form, it is clear that only agents who are capable of making hypothetical judgments and subsuming particulars under general concepts can be said to make commitments, or said to be either consistent or inconsistent.

This analysis again provides us with an illuminating account of Robert’s case. On Kant’s analysis, disorganized speech is due to an impairment of the understanding and the power of judgment. Since agents must possess these faculties in order to be able to make commitments, it is expected that Robert’s actions will not express any policies. Indeed, it would be farfetched to assume that Robert adopted a policy of destroying television sets after closing hours or that he decided to destroy the television sets ‘only for this once.’ Robert’s actions thus do not express any attitudes. Since Robert’s actions do not express any intentions or attitudes, they express neither good will nor a lack thereof. And if we blame agents because their actions express a lack of good will, we can safely conclude that, at least as long as they lack the relevant capacities, agents such as Robert are exempted from moral blame.

## Conclusion

In this paper, I have offered a Kantian answer to the question whether and why it would be inappropriate to blame people suffering from disorders that fall within the schizophrenia spectrum. Notably, the Kantian picture suggests that even if we focus exclusively on these disorders, there is no unified answer to that question. Patients who suffer from delusions or hallucinations are exculpated on different grounds than patients with formal thought disorder. Specifically, I have argued that the former group of patients should be exempted from doxastic responsibility because they lack the ability to enter into a mutual exchange of epistemic reasons and to revise their beliefs in the light of evidence and proof. On the assumption that they are practically rational and have the required normative abilities, these patients are still members of the moral community and subject to a demand of good will. They are exonerated from blame only on the condition that their actions would be morally permitted or excused had their beliefs been true. I have argued that the latter group of patients should be exempted from moral responsibility because by being unable to form intentions and adopt policies, they fail to satisfy the requirements of agency.

## Footnotes

1 The terms ‘excuses’ and ‘exemptions’ are coined by Watson (1987).

2 Without endorsing it, Korsgaard notes, ‘if we […] regard people as free agents […] then it seems as if we must treat them as transcendentally free and so as completely responsible for each and every action, no matter what sorts of pressures they may be under’ (1996a, 205).

3 Recent collections of essays on Kant’s anthropology contain no essay on mental disorder. See Jacobs (2007); Heidemann (2011) and Cohen (2014). The most important monographs on Kant’s anthropology do not contain a significant analysis of the topic either. See Munzel (1999); Louden (2000, 2011); Frierson (2003); Wilson (2006); Sturm (2009) and Cohen (2009). Two essays by Frierson (2009a, b) are notable exceptions, but Frierson does not extensively treat questions of responsibility.

4 All references to Kant’s works are to the *Cambridge Edition of the Works of Immanuel Kant*. Numbers refer to the volumes and pages of the standard edition of Kant’s works by the Royal German Academy of the Sciences. Numbers in references to the *Critique of Pure Reason* refer to the page numbers of the original A and B editions. I will use the following abbreviations:

A/B: *Critique of Pure Reason*, Kant (1998).
Anth: *Anthropology from a Pragmatic Point of View*, in Kant (2007).
DMC: *On the Philosopher’s Medicine of the Body*, in Kant (2007).
GMS: *Groundwork of the Metaphysics of Morals*, in Kant (1996).
KpV: *Critique of Practical Reason*, in Kant (1996).
KU: *Critique of the Power of Judgment*, Kant (2000).
MS: *Metaphysics of Morals*, in Kant (1996).
Prol: *Prolegomena to any Future Metaphysics*, in Kant (2002).
VKK: *Essay on the Maladies of the Head*, in Kant (2007).
V-M_1_: *Lectures on Metaphysics* *L*_1_ (Heinze), in Kant (1997b).
V-MS: *Lectures on the Metaphysics of Morals* (Vigilantius), in Kant (1997a).
WA: An Answer to the Question: ‘What is Enlightenment?’, in Kant (1991).

5 The definition of mental disorder in the current fifth edition of the *Diagnostic and Statistical Manual of Mental Disorders* does not contain the phrase ‘loss of freedom’ (cf. American Psychiatric Organization 2013, 20). Presumably, however, the phrase is omitted merely for the sake of clarity. Both the American Psychiatric Organization 1994 and the American Psychiatric Organization 2013 definition of mental disorder mention the concept of disability. Since disability already implies a loss of freedom, a group of influential scholars has judged that the definition of mental disorder could be simplified by omitting the phrase (cf. Stein et al. 2010, 1762). In light of this, the association between mental disorder and a loss of freedom, as stated in American Psychiatric Organization 1994, is still relevant.

6 Wallace (1994, 154ff.) provides a pertinent example of the generalist approach. Wallace takes the mentally disordered to be exempted from moral responsibility because they lack what he calls ‘the powers of reflective self-control.’ The impairment of these powers would explain why mental disorders as diverse as depression, paranoid psychosis, obsessive-compulsive disorder and anti-social personality disorder qualify for exemption.

7 I adopt this distinction between justifications and excuses from Austin (1956).

8 The idea that agents are exempted from moral responsibility because they are not capable of being morally addressed has been developed by Watson (1987) and taken up by Wallace (1994); Darwall (2006) and McKenna (2011).

9 For example, Wallace (1994) distinguishes between ‘blameworthiness’ and ‘accountability conditions.’

10 I adopt Wallace’s typology of the excuses (1994, 136ff.). In Wallace’s terminology, successful excuses show that one ‘did not really do *x*.’ Since there are cases in which (at least in some sense) an agent did *x* and is excused for *x*, a lot hinges on what exactly Wallace means by ‘really’ doing *x*. To avoid confusion, I use the Davidsonian terminology of actions being ‘intentional under a description.’

11 As a psychiatrist, Spitzer (1990) also argues that Kant’s psychopathology is relevant to the understanding of the symptoms of schizophrenia. However, his analysis focuses exclusively on so-called ‘passivity experiences.’

12 See Kant’s *On the Philosopher’s Medicine of the Body* (DMC 15: 945, 947).

13 Foucault (2006, 123, 126) criticizes Kant for his claim that in order to determine whether the agent was ‘mad’ at the time of his action, the judge must refer him to the philosophical rather than the medical faculty (cf. Anth 7: 213). However, I think this claim is not implausible if we take into account that in Kant’s time the discipline of empirical psychology was still part of the philosophical faculty. To be sure, medical data is relevant for questions about responsibility inasmuch it provides evidence for the presence or absence of certain psychological states and capacities. But Kant thought (rightly, I think) that the evidence provided by the ‘physicians and physiologists’ of his time was not yet reliable (Anth 7: 214).

14 The term ‘dementia’ had a much broader meaning in the seventeenth and eighteenth century than it has today and was not yet particularly associated with a group of people of a certain age (Berrios 1987).

15 For this formal difference between these two kinds of representations, see Kant’s discussion of dreams in the *Prolegomena* (Prol 4: 290f.).

16 For a Kantian explanation of the phenomenon of thought insertion, see Chadwick (1994) and Young (2006).

17 This example is taken from a case report by Pfuhlmann et al. (1998). The translation is mine.

18 Command hallucinations, a class of auditory hallucinations, will play an important role in one of the cases I discuss in ‘Epistemic Incapacity’ section.

19 Kant claims that with regard to the mentally deranged duties of love and beneficence take precedence over duties of respect. Whereas duties of respect require us to respect the other’s choices, duties of love require us to provide the other with appropriate assistance. Arguing that normally there is an unresolvable tension between these duties, Kant claims that with regard to young children and the mentally deranged duties of love take priority: ‘I cannot do good to anyone in accordance with *my* concepts of happiness (except to young children and the insane)’ (MS 6: 454).

20 Implicit in every maxim is an end and a motive: one performs action *A* in order to attain some end *E* and from some motive *M*. These can be made explicit by counterfactual questioning. This strategy is developed by O’Neill (1975).

21 For an overview of this discussion, see Gressis (2010a, b).

22 According to this definition, my disposition to fall if you push me down the stairs is not an attitude, whereas my disposition to step on the gas if a traffic light turns to amber is.

23 In his lectures, Kant gives the example of someone who due to a ‘dizzy spell’ pushed another person into the water. He argues that this agent should not be blamed because the cause of the event ‘was merely physical and a matter of natural necessity; it rested on no originative cause in the agent’ (V-MS 27: 559).

24 Those who want some assurance that Kant actually thinks of coercion and necessity as excusing conditions may want to take a look at his discussion of the alleged ‘right of necessity’ in the *Doctrine of Right*. There Kant claims that while ‘there could be no necessity that would make what is wrong conform to law’ (MS 6: 236), the act of shoving an innocent person off a floating plank in order to save oneself from drowning is ‘*unpunishable*’ (MS 6: 235f.). In short, necessity is not a justification but an excuse. It must be noted that Kant here argues that necessity is a legal excuse. He is silent on whether it also constitutes a moral excuse.

25 The examples are inspired by Parfit (2011, Ch. 7).

26 By contrast, Herman would presumably hold that Joe’s action is morally permissible. The reason is that she believes that on the Kantian view ‘good intentions plus adequate care *are* enough’ to make an action morally permissible (1993, 98). She then tries to account for the intuitive moral difference between cases such as that of Jane and Joe in terms of the responses called for by negative outcomes. I believe that Kant takes moral permissibility to be more objective than Herman suggests. This is confirmed by a passage from the *Doctrine of Virtue*, where Kant asserts that ‘I can indeed be mistaken at times in my objective judgment as to whether something is a duty or not’ (MS 6: 401). Importantly, he adds that if someone acted conscientiously, ‘then as far as guilt or innocence is concerned nothing more can be required of him. It is incumbent upon him only to enlighten his *understanding* in the matter of what is or is not duty’ (MS 6: 401). This suggests that although the combination of good intentions and adequate care is sufficient for moral exculpation, it is not for moral permissibility. This is in keeping not only with the criterion of permissibility that I have proposed, but also with the analysis of the excuse of inadvertence that I develop in what follows.

27 The assumption is not entirely artificial. There are case reports of so-called ‘pure’ cases of paranoid schizophrenia, patients whose mental state is normal except for some encapsulated delusion. See Walston et al. (2000).

28 Neither does Kant. In particular, he claims that patients suffering from *dementia* and *vesania* are not dangerous (Anth 7: 215f.).

29 This case is adapted from Walston et al. (2000).

30 This case is adapted from Driscoll et al. (1991).

31 This case is adapted from Byrne et al. (2006).

32 This case is adapted from Elliott (1996, 108).
